# Association between Seminal Oxidation-Reduction Potential and Sperm DNA Fragmentation—A Meta-Analysis

**DOI:** 10.3390/antiox11081563

**Published:** 2022-08-12

**Authors:** Manesh Kumar Panner Selvam, Saradha Baskaran, Samantha O’Connell, Wael Almajed, Wayne J. G. Hellstrom, Suresh C. Sikka

**Affiliations:** 1 Department of Urology, School of Medicine, Tulane University, New Orleans, LA 70112, USA; 2 Office of Academic Affairs and Provost, Tulane University, New Orleans, LA 70112, USA

**Keywords:** oxidative stress, male infertility, oxidation-reduction potential, sperm DNA fragmentation, meta-analysis

## Abstract

Seminal oxidative stress and sperm DNA damage are potential etiologies of male factor infertility. The present study aims to evaluate the relationship between oxidation-reduction potential (ORP), a measure of oxidative stress, and sperm DNA fragmentation (SDF) by conducting a systematic review and meta-analysis of relevant clinical data. A literature search was performed according to the Preferred Reporting Items for Systematic Reviews and Meta-Analyses (PRISMA) guidelines. The COVIDENCE tool was used to screen and identify studies evaluating seminal ORP and SDF. Studies (*n* = 7) that measured seminal ORP and SDF of 3491 semen samples were included in the analysis. The fixed-effects model revealed a significant pooled correlation coefficient (r = 0.24; *p* < 0.001) between seminal ORP and SDF. Furthermore, subgroup analyses indicated that the pooled correlation coefficient between ORP and sperm chromatin dispersion (SCD) assay was less than other SDF assays (0.23 vs. 0.29). There was a moderate level of heterogeneity (I^2^ = 42.27%) among the studies, indicating a lack of publication bias. This is the first meta-analysis to reveal a positive correlation between seminal ORP and SDF. Furthermore, this study indicates the role of oxidative stress in the development of sperm DNA damage and thus warrants prospectively exploring the clinical value of these sperm function tests.

## 1. Introduction

Infertility affects 8–12% of couples of reproductive age, with the male component being implicated as the causative factor in up to 50% of cases [1]. Evidence implicates the role of oxidative stress in the pathophysiology of male infertility [2,3]. About 30–80% of infertile men have elevated levels of seminal reactive oxygen species (ROS) [4]. At physiological levels, ROS act as regulatory molecules and play a pivotal role in sperm functions such as capacitation, acrosome reaction, hyperactivation, and fertilization [5,6]. However, when concentrations of ROS exceed the scavenging capacity of seminal antioxidants referred to, oxidative stress status (OSS) can cause damage to cellular components, which leads to poor sperm quality [5,7]. Therefore, maintaining a subtle balance between the seminal oxidants and reductants is crucial for redox homeostasis. Recently, a novel method for measuring oxidation-reduction potential (ORP) in semen has been introduced to evaluate seminal oxidative stress based on the redox balance [8]. Scientometric analysis of oxidative stress in male reproductive research reveals a number of publications on studies using ORP over a short period of time, suggesting the emergence of ORP as a future direct assay to measure oxidative stress in semen samples [9]. In addition, ORP efficiently assesses oxidative stress by utilizing small amounts of fresh or frozen semen samples with readily available results [10], thereby making this test attractive for wider application in the clinical and research setting [11].

Sperm DNA integrity is vital for fertilization and embryo development [12]. One of the detrimental effects of oxidative stress on sperm is the induction of SDF [5,13,14]. Studies have substantiated the strong association between high seminal levels of ROS and sperm DNA damage [13,15]. Although oxidative stress is one of the key mechanisms underlying sperm DNA damage, the exact association between ORP, a direct indicator of oxidative stress, and sperm DNA damage in infertile men has not been investigated systematically. Therefore, the main objective of this study is to evaluate the relationship between ORP and sperm DNA damage by conducting a systematic review and meta-analysis of relevant scientific literature.

## 2. Materials and Methods

### 2.1. Search Strategy

**Figure 1:**
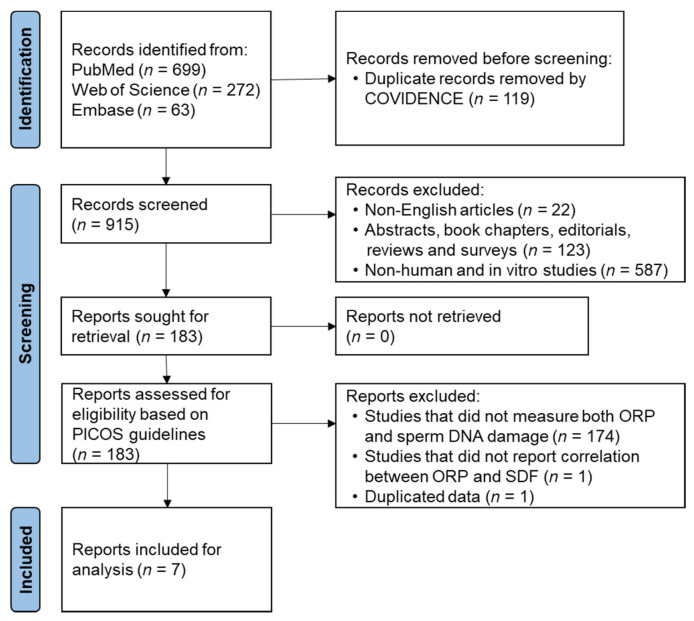
Flowchart of study identification and selection. ORP: oxidation-reduction potential, PICOS: population, intervention, comparison, outcome, and study design, SDF: sperm DNA fragmentation.

Suitable keywords were used to retrieve articles reporting both seminal oxidative stress and sperm DNA damage from databases such as PubMed, Web of Science, and Embase (Table S1). In August 2021, a systematic review of literature (Figure 1) search was conducted according to the Preferred Reporting Items for Systematic Reviews and Meta-Analyses (PRISMA) guidelines [16]. The COVIDENCE online tool (Veritas Health Innovation, Melbourne, Australia) was used to screen and identify studies evaluating both seminal ORP and SDF. Duplicate articles retrieved from different databases were removed, and the remaining articles were independently screened for title, abstract, and keywords by two authors (MKPS and SB) to exclude irrelevant studies based on inclusion and exclusion criteria (Table S2). Full-text articles were screened for eligibility based on the Population, Intervention, Comparison, Outcome, and Study design (PICOS) tool that excludes irrelevant articles and increases the reproducibility of the results. Data extraction from selected articles was performed independently by two investigators (MKPS and SB), and any discrepancies were settled by a senior author. If results of a study are published more than once, only those with the most complete and up-to-date information were included in the analysis.

### 2.2. Quality Assessment of Individual Studies

A modified Newcastle-Ottawa Scale was used to assess the quality of studies included in this analysis (Table S3). Two investigators (MKPS and SB) independently evaluated all eligible articles. Each study was assigned a score ranging from 0 to 10, and its quality was indicated by scores: 0–3 indicates poor quality, 4–7 indicates fair quality, and 8–10 indicates good quality.

### 2.3. Meta-Analysis

Statistical analysis was conducted by using STATA statistical software version 12.0 (Stata Corp LP, College Station, TX, USA). The Pearson correlation coefficient between seminal ORP and sperm DNA damage served as the effect size for synthesis. The correlation for each study was weighted by the inverse of its variance. Correlation coefficients and their standard errors were transformed to the Fisher’s z scale for meta-analysis. All results were converted back to correlations for reporting. Fisher’s z-transformed correlations were pooled by using the fixed-effects model, due to insignificant heterogeneity among effect sizes. Q and I^2^ statistics were used to assess heterogeneity of effect sizes. A forest plot was produced to visually assess the correlation and corresponding 95% confidence intervals across studies. A subgroup analysis was conducted according to the SDF method to test whether the SDF method impacted the correlation between seminal ORP and sperm DNA damage. The SDF method was separated into two groups (a) sperm chromatin dispersion (SCD) and (b) other SDF methods that included terminal deoxynucleotidyl transferase (dUTP) nick end labelling (TUNEL) and sperm chromatin structure assay (SCSA). Possible publication bias was assessed by visual inspection of the funnel plot and the trim-and-fill method [17].

## 3. Results

The search strategy identified a total of 921 articles after removing duplicates (*n* = 119) (Figure 1). Based on inclusion and exclusion criteria, title, abstract and keywords, screening revealed 183 articles eligible for full-text retrieval. From these articles, seven studies were included in this meta-analysis based on the PICOS framework.

### 3.1. Characteristics of Eligible Studies and Methodological Quality

Seven studies that measured both seminal ORP and SDF of 3491 semen samples from men attending fertility clinics were included in the meta-analysis. The characteristics of the seven studies are presented in Table 1. Of these, the majority of the studies (*n* = 5) were of good quality according to the modified Newcastle-Ottawa Scale, although others (*n* = 2) showed some concerns and were of fair quality (Table 1 and Table S4).

**Table 1:** Details of the original studies evaluated both ORP and SDF in semen samples.

Studies (Author, Year)	Study Type	Sample Size	SDF Methods	ORP vs SDF (Correlation Coefficient)	*p*-Value	* Study Quality (Maximum Score = 10 Stars)
Arafa et al., 2019 [18]	Retrospective	659	SCD	0.264	<0.0001	9
Arafa et al., 2020 [19]	Retrospective	1068	SCD	0.218	<0.0001	9
Garcia-Segura et al., 2020 [20]	Cross-sectional	42	TUNEL	0.160	NS	6
			Alkaline comet	0.125	NS	
Gill et al., 2021 [21]	Cross-sectional	167	SCD	0.364	<0.000001	8
Homa et al., 2019 [22]	Cross-sectional	47	SCSA	0.23992	0.1043	7
Majzoub et al., 2018–1 [23]	Prospective case-control	1168	SCD	0.222	0.001	10
Majzoub et al., 2018–2 [23]	Prospective case-control	100	SCD	0.004	NS	
Tanaka et al., 2020 [24]	Cross-sectional	240	SCSA	0.320075	<0.001	8

NR: not reported, NS: non-significant, ORP: oxidation-reduction potential, SDF: sperm DNA fragmentation, SCD: sperm chromatin dispersion, SCSA: sperm chromatin structure assay, TUNEL: terminal deoxynucleotidyl transferase (dUTP) nick end labeling. * A modified Newcastle-Ottawa Scale was used for assessment of study quality.

### 3.2. Correlation between ORP and SDF

#### 3.2.1. Pooled Correlation

**Figure 2:**
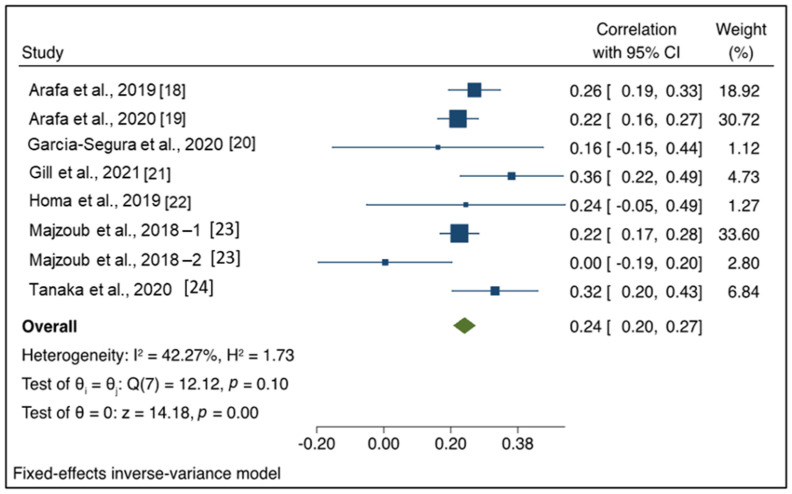
Forest plot of correlation coefficient between seminal oxidation-reduction potential and sperm DNA fragmentation.

The fixed-effects model revealed that the pooled correlation coefficient between seminal ORP and SDF was 0.24 (95% CI: 0.20, 0.27), as presented in Figure 2. The Q and I2 statistics indicated insignificant heterogeneity (I2 = 42.27%, *p* = 0.10). The z value of the test ES = 0 was 14.18, *p* < 0.001. Therefore, the pooled effect size estimation was significant (*p* < 0.05).

#### 3.2.2. Subgroup Analysis

**Figure 3:**
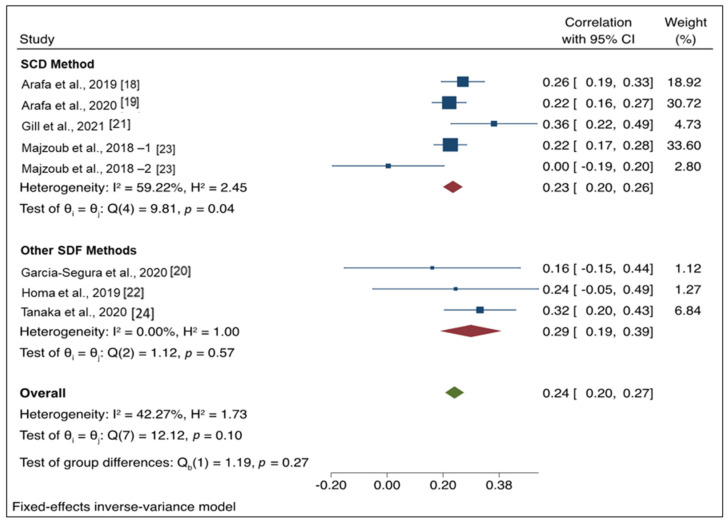
Forest plot for subgroup analysis of correlation coefficient between seminal oxidation-reduction potential and different sperm DNA fragmentation methods.

Results of the subgroup analysis are presented in Figure 3. Five studies were included in the SDF method subgroup, and three studies were included in the other methods subgroup. For studies using the SCD method, the pooled correlation coefficient was 0.23 (95% CI: 0.2, 0.26), whereas the pooled correlation coefficient for other SDF methods was 0.29 (95% CI: 0.19, 0.39). The test of group differences indicated no significant differences between groups (Q_b_ (1) = 1.19, *p* = 0.27). There was a moderate level of heterogeneity (I2 = 42.27%) among the studies; however, this was not significant (*p* = 0.10).

### 3.3. Publication Bias

**Figure 4:**
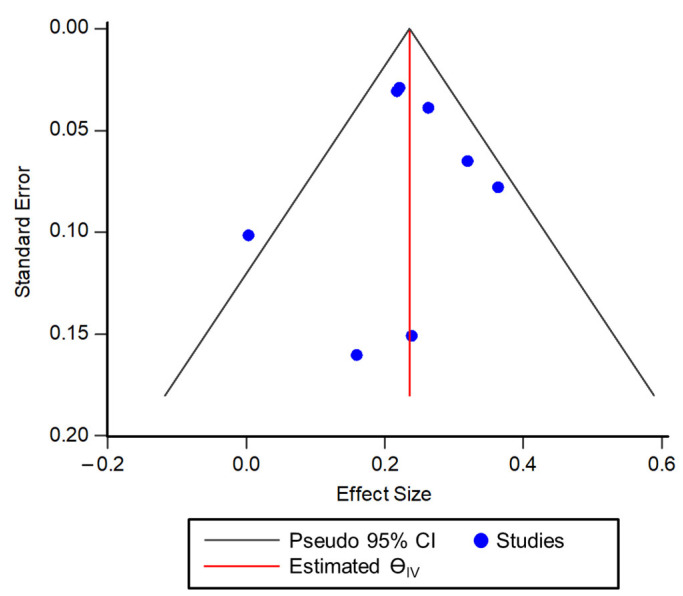
Funnel plot for visual depiction of publication bias among the studies included in the meta-analysis.

Visual inspection of the Funnel plot indicated a lack of publication bias, as there was symmetry (Figure 4). The distribution of correlation values for each study was approximately symmetrical around the pooled correlation estimate between seminal ORP and SDF. The trim-and-fill method revealed no imputed studies, further indicating a lack of publication bias.

## 4. Discussion

The evaluation of male factor fertility potential is primarily based on physical examination and conventional semen analysis, as recommended by the World Health Organization (WHO) [25]. However, a major drawback of semen analysis is that conventional sperm parameters are crude indicators of male fertility potential, and the reference ranges for these parameters were set based on a population of fertile couples who had succeeded in achieving a natural conception. Therefore, there is a need to explore specialized biomarkers that accurately assess both male infertility and fertility potential. The recent WHO manual highlights the significance of advanced examinations, such as seminal oxidative stress and sperm chromatin assessment in understanding the enigmatic mechanisms underlying human sperm dysfunction [25]. A few recent studies have proposed that the imbalance in oxidation-reduction reactions associated with oxidative stress and subsequent sperm DNA damage could hamper male fertility [12,13,14]. However, the association between ORP and sperm DNA damage has not been examined systematically. Our meta-analysis explicitly revealed a significant relationship between ORP and sperm DNA damage, indicating the role of oxidative stress in sperm DNA damage and therefore suggesting potential clinical value of these advanced parameters in evaluating male fertility status.

Oxidative stress plays a major role in the pathophysiology of male infertility caused by various clinical, environmental, and lifestyle factors [4,26,27]. An imbalance between generation of ROS and the antioxidant scavenging system results in seminal oxidative stress monitored by OSS, and the associated oxidative damage to proteins, lipids, and DNA can, in turn, compromise the structural and functional integrity of spermatozoa [26,28]. Standard direct assays to evaluate seminal oxidative stress involve measurement of ROS levels in semen, and indirect assays involve assessment of individual/total antioxidants or oxidized products (i.e., malondialdehyde) and modified base (i.e., 8-hydroxy-2′-deoxyguanosine) [29]. On the other hand, the ORP test measures the oxidative stress in semen samples based on dynamic redox balance [11]. The application of ORP in clinical research as a tool to evaluate seminal oxidative stress is increasing due to it being a simple and rapid test [11]. A cut-off value of 1.34 mV/10^6^ sperm/ ml has been established based on a multicenter study to differentiate men with normal and abnormal semen parameters [4,30]. Men with higher ORP levels than the reference value indicates a state of seminal oxidative stress that may increase sperm DNA damage.

ORP and sperm DNA damage were correlated with poor sperm parameters in men with infertility [30,31,32,33,34,35]. Sperm DNA is assessed for both its integrity as well as degree of fragmentation. Among many different methods for assessing sperm DNA damage, SDF assays are the most commonly used in andrology laboratories. Several guidelines highlight the clinical utility of SDF tests in evaluating male infertility [36,37,38]. Moreover, recent studies have also evaluated the association between ORP and SDF [19,39]. The current meta-analysis revealed a positive correlation between ORP and SDF with a lack of publication bias. However, some discrepancies exist among the published studies, with results reporting either association or no association between ORP and SDF values in a clinical setup [20,23,24]. This discrepancy may be attributed to differences of SDF protocols, different enzymes and slide kit suppliers, and different methods of DNA damage detection among those studies. Our meta-analysis results indicate that TUNEL and SCSA values correlate well with ORP results, as compared to SCD test values. However, it is also important to highlight that most studies included in our meta-analysis were retrospective or cross-sectional. Furthermore, there is also a need to clarify whether the type of infertility (primary, secondary, or idiopathic) influences the ORP and SDF test results. Hence, prospective studies with proper patient selection are needed to provide better insight regarding the association between SDF and ORP test results.

The current meta-analysis has certain limitations. Some studies had low sample size (or) reported variation among the subjects (or) weak correlation between ORP and SDF (or) lack of uniformity among assays used for DNA damage detection. In addition, there is a lack of information in regard to infertility duration, treatment, hormonal levels, history of genitourinary inflammation (GUI), lifestyle factors, etc. All these are likely to affect the SDF and ORP values. Despite these limitations, our findings are conclusive due to the inclusion of >3000 patients with male factor infertility issues. Furthermore, a majority of the studies included are of good quality according to the modified Newcastle-Ottawa Scale. A major strength of our meta-analysis is that there is a moderate degree of heterogeneity among the studies with no publication bias.

## 5. Conclusions

This study is the first meta-analysis to evaluate the relationship between the newer seminal oxidative stress markers, mainly ORP and sperm DNA damage. The analysis revealed a positive correlation between seminal ORP and SDF. Furthermore, this study indicates the role of oxidative stress in the development of sperm DNA damage and thus warrants prospectively exploring the clinical application of these tests as related to male infertility diagnosis and better treatment modalities.

## Data Availability

Data is contained within the article.

## Supplementary Materials

The following supporting information can be downloaded at: https://www.mdpi.com/article/10.3390/antiox11081563/s1, Table S1: Search terms or keywords used to search different databases to identify relevant articles; Table S2: Inclusion and exclusion criteria used to identify the relevant articles for meta-analysis; Table S3: A modified Newcastle-Ottawa Scale for assessment of study quality; Table S4: Grading of studies included for meta-analysis using a modified Newcastle-Ottawa Scale for assessment of study quality.
