# Supplementary material for: Association between Seminal Oxidation-Reduction Potential and Sperm DNA Fragmentation—A Meta-Analysis
Source: Antioxidants (Basel). 2022 Aug 12;11(8):1563. doi: 10.3390/antiox11081563 (PMC9404741; doi:10.3390/antiox11081563)
Supplement: Supplementary file 1 [file antioxidants-11-01563-s001.zip › antioxidants-1836141-supplementary/Table S1.pdf]

Table S1: Search terms or keywords used to search different databases to identify relevant articles

**PubMed (search date: 8/11/2021)**
(((Infertility, Male[Mesh] OR "male infertility"[tw] OR "male sterility"[tw] OR "male subfertility"[tw] OR ((Male[Mesh] OR Men[Mesh] OR male[tw] OR males[tw] OR male*[tw] OR men[tw] OR men's[tw] OR man[tw]) AND (Infertility[Mesh] OR sterility[tw] OR steril*[tw] OR subfertility[tw] OR subfertil*[tw] OR infertil*[tw] OR sub-fertility[tw] OR sub-fertil*[tw] OR "sub fertility"[tw] OR "sub fertil*"[tw]))) OR (Spermatozoa[Mesh] OR "Ejaculation"[Mesh] OR "Semen"[Mesh] OR "Penis/metabolism"[Mesh] OR "sperm motility"[Mesh] OR sperm[tw] OR spermatozoa[tw] OR spermatozoon[tw] OR sperms[tw] OR sperm*[tw] OR "sperm cell"[tw] OR (sperm[tw] AND cell*[tw]) OR semen[tw] OR (sperm[tw] AND motility[tw]) OR ejaculation[tw] OR ejaculat*[tw])) AND (Oxidation-Reduction[MeSH Terms] OR ORP[tw] OR "oxidation-reduction potential"[tw] OR "oxidation reduction potential"[tw] OR (oxidation-reduction[tw] AND potential[tw]) OR (oxidation[tw] AND reduction[tw] AND potential[tw]) OR oxidation-reduction[tw] OR "oxidation reduc*"[tw])) AND (Chromatin[MeSH Terms] OR "DNA Fragmentation"[MeSH Terms] OR "DNA damage"[tw] OR (DNA[tw] AND damage[tw]) OR "DNA fragmentation"[tw] OR (DNA[tw] AND fragment*[tw]) OR "sperm dna damage"[tw] OR "sperm dna fragmentation"[tw] OR "chromatin dispersion"[tw] OR (chromatin[tw] AND dispersion[tw]) OR (chromatin[tw] AND dispers*[tw]) OR "sperm chromatin dispersion"[tw] OR "sperm damage"[tw] OR (sperm[tw] AND damage[tw]) OR "sperm integrity"[tw] OR "DNA integrity"[tw] OR (dna[tw] AND integrity[tw]) OR (sperm[tw] AND integrity[tw]))

**Embase (search date: 8/23/2021)**
(('male infertility'/exp OR 'male infertility':ab,ti,kw OR 'male sterility'/exp OR 'male sterility':ti,ab,kw OR 'male subfertility'/exp OR 'male subfertility':ti,ab,kw OR 'male'/exp OR 'male':ti,ab,kw OR 'males':ti,ab,kw OR 'men':ti,ab,kw OR 'man':ti,ab,kw) AND ('infertility'/exp OR 'infertility':ti,ab,kw OR 'fertility disorder':ti,ab,kw OR 'infecundity':ti,ab,kw OR 'primary infertility':ti,ab,kw OR 'secondary infertility':ti,ab,kw OR 'sexual sterility':ti,ab,kw OR 'sterility'/exp OR 'sterility':ti,ab,kw OR 'sterile':ti,ab,kw OR 'subfertility'/exp OR 'subfertile':ti,ab,kw OR 'subfertility':ti,ab,kw OR 'sub-fertile':ti,ab,kw OR 'sub-fertility':ti,ab,kw OR 'sub fertility':ti,ab,kw OR 'sub fertile':ti,ab,kw) OR 'spermatozoon'/exp OR 'spermatozoon':ti,ab,kw OR 'spermatic cell':ti,ab,kw OR 'spermatozoa':ti,ab,kw OR 'spermatozoid':ti,ab,kw OR 'ejaculation'/exp OR 'ejaculation':ti,ab,kw OR 'seminal discharge':ti,ab,kw OR 'sperm release':ti,ab,kw OR 'sperm'/exp OR 'sperm':ti,ab,kw OR 'sperm cell':ti,ab,kw OR 'ejaculate':ti,ab,kw OR 'semen':ti,ab,kw OR 'sperm utilization':ti,ab,kw OR 'sperma':ti,ab,kw OR 'spermatozoon motility'/exp OR 'spermatozoon motility':ti,ab,kw OR 'semen mobility':ti,ab,kw OR 'semen motility':ti,ab,kw OR 'seminal motility':ti,ab,kw OR 'sperm mobility':ti,ab,kw OR 'sperm motility':ti,ab,kw OR 'spermatic motility':ti,ab,kw OR 'spermatozoa mobility':ti,ab,kw OR 'spermatozoa motility':ti,ab,kw OR 'spermatozoal mobility':ti,ab,kw OR 'spermatozoal motility':ti,ab,kw OR 'spermatozoan mobility':ti,ab,kw OR 'spermatozoan motility':ti,ab,kw OR 'spermatozoid motility':ti,ab,kw OR 'spermatozoon mobility':ti,ab,kw) AND ('chromatin'/exp OR 'chromatin':ti,ab,kw OR 'chromatin disper*':ti,ab,kw OR 'sperm chromatin dispersion':ti,ab,kw OR 'dna damage'/exp OR 'dna damage':ti,ab,kw OR 'sperm dna damage':ti,ab,kw OR 'sperm damage':ti,ab,kw OR 'sperm integrity':ti,ab,kw OR 'dna integrity':ti,ab,kw OR 'dna fragmentation'/exp OR 'dna fragmentation':ti,ab,kw OR 'dna fragment*':ti,ab,kw OR 'sperm dna fragmentation':ti,ab,kw) AND ('oxidation reduction potential'/exp OR 'oxidation reduction potential':ti,ab,kw OR 'oxidation potential':ti,ab,kw OR 'oxidative potential':ti,ab,kw OR 'oxido reduction potential':ti,ab,kw OR 'oxidoreduction potential':ti,ab,kw OR 'redox potential':ti,ab,kw OR 'redoxpotential':ti,ab,kw OR 'oxidation reduc*':ti,ab,kw OR 'orp':ti,ab,kw)

Web of Science (search date: 8/24/2021)
(male infertility) OR (male infertil*) OR (male steril*) OR (male sub-fertil*) OR (male subfertil*) OR (male sub fertil*) OR (infertil* men) OR (sterile men) OR (sub fertil* men) OR (subfertil* men) OR (sub-fertil* men) OR (sperm*) OR (semen) OR (ejaculat*) OR (sperm cell) OR (sperm motility) OR (sperm mobility) (All Fields) and (chromatin) OR (dna fragment*) OR (dna damage) OR (chromatin dispers*) OR (sperm damage) OR (sperm integrity) OR (dna integrity) OR (sperm chromatin dispers*) OR (sperm dna fragment*) OR (sperm dna damage) (All Fields) and (oxidation reduction potential) OR (ORP) OR (oxidation reduction) OR (oxidation reduc*) OR (redox potential) OR (oxidoreduction potential) (All Fields)
