# Supplementary material for: Association between Seminal Oxidation-Reduction Potential and Sperm DNA Fragmentation—A Meta-Analysis
Source: Antioxidants (Basel). 2022 Aug 12;11(8):1563. doi: 10.3390/antiox11081563 (PMC9404741; doi:10.3390/antiox11081563)
Supplement: Supplementary file 1 [file antioxidants-11-01563-s001.zip › antioxidants-1836141-supplementary/Table S2.pdf]

Table S2: Inclusion and exclusion criteria used to identify the relevant articles for meta-analysis

**Inclusion Criteria**
Clinical trials and observational studies in adult males
Measuring seminal oxidative stress using ORP and sperm DNA damage/ integrity using any assay
Semen analysis performed based on WHO 5th Edition (2010 guidelines)
Studies reporting correlation between ORP and Sperm DNA damage
Published articles; Abstracts; Conference Proceedings; Pre-Print Manuscripts; Dissertations
**Exclusion Criteria**
Animal studies, *in vitro* studies, *in silico* studies
Meta-analysis, systematic reviews
Case reports studies
Non-English text articles
