# Supplementary material for: Association between Seminal Oxidation-Reduction Potential and Sperm DNA Fragmentation—A Meta-Analysis
Source: Antioxidants (Basel). 2022 Aug 12;11(8):1563. doi: 10.3390/antiox11081563 (PMC9404741; doi:10.3390/antiox11081563)
Supplement: Supplementary file 1 [file antioxidants-11-01563-s001.zip › antioxidants-1836141-supplementary/Table S3.pdf]

Table S3: A modified Newcastle-Ottawa Scale for assessment of study quality

Category	Scoring Criteria
**Selection**<br>(Maximum score: 2 stars)	**Representativeness of the cohort**
	Truly representative (1 star)
	Somewhat representative (1 star)
	Highly selected group (No star)
	No description (No star)
	**Ascertainment of exposure**
	Secure record (1 star)
	Directly measured (1 star)
	Written self-report (No star)
	No description (No star)
**Comparability**<br>(Maximum score: 2 stars)	**Comparability of cohort, controlling of confounders**
	Controls of key confounders and related factors (eg. Testicular cancer, genetic defects, smoking, environmental exposure, etc.) (1 star)
	Uncontrolled for confounders (No star)
	**Baseline characteristics**
	Demographic and clinical characteristics of study population presented (1 star)
	No description (No star)
**Outcome**<br>(Maximum score: 6 stars)	**Assessment of outcome**
	Record linkage (1 star)
	Self-report (No star)
	No description (No star)
	**Assessment of oxidative stress and Sperm DNA damage**
	Assessment of ORP (1 star)
	Assessment of SDF (1 star)
	No description (No star)
	**Adequacy of sample size**
	**Number of samples analyzed ≥100**
	Number of samples analyzed equal to the number of subjects in the group (1 star)
	More than or equal to 80% of the sample analyzed or description for the exclusion provided (1 star)
	Less than 80% of the sample analyzed and no description for the exclusion (No star)
	Number of samples analyzed <100 (No star)
	No description (No star)
	**Correlation between ORP and SDF**
	Fertile men/ donor group (1 star)
	Infertile men (1 star)
	No description (No star)
	Others (No star)

Good quality: 2/4 stars for selection, 1/2 stars for comparability, 5/6 stars for outcome
Fair quality: 1/4 stars for selection, 1/2 stars for comparability, 4/6 stars for outcome
Poor quality: 0/4 stars for selection, 0/2 stars for comparability, 3/6 stars for outcome
