# Supplementary material for: Association between Seminal Oxidation-Reduction Potential and Sperm DNA Fragmentation—A Meta-Analysis
Source: Antioxidants (Basel). 2022 Aug 12;11(8):1563. doi: 10.3390/antiox11081563 (PMC9404741; doi:10.3390/antiox11081563)
Supplement: Supplementary file 1 [file antioxidants-11-01563-s001.zip › antioxidants-1836141-supplementary/Table S4.pdf]

Table S4: Grading of studies included for meta-analysis using a modified Newcastle-Ottawa Scale for assessment of study quality

Study	Selection [Maximum Score =2 stars]		Comparability [Maximum Score =2 stars]		Outcome [Maximum Score =6 stars]				Total Score (Maximum Score =10 stars)
	**1) Representativeness of the cohort [Maximum Score =1 star]**	**2) Ascertainment of exposure [Maximum Score =1 star]**	**Comparability of cohort, controlling of confounders [Maximum Score =1 star]**	**Baseline characteristics [Maximum Score =1 star]**	**1) Assessment of outcome [Maximum Score =1 star]**	**2) Assessment of oxidative stress and sperm DNA damage [Maximum Score =2 stars]**	**3) Adequacy of sample size [Maximum Score =1 star]**	**4) Correlation between ORP and SDF [Maximum Score =2 stars]**	
	a) Truly representative (1 star)<br>b) Somewhat representative (1 star)<br>c) Highly selected group (No star)<br>d) No description (No star)	a) Secure record (1 star)<br>b) Directly measured (1 star)<br>c) Written self-report (No star)<br>d) No description (No star)	a) Controls of key confounders and related factors (eg. Testicular cancer, genetic defects, smoking, environmental exposure, etc.) (1 star)<br>b) Uncontrolled for confounders (No star)	a) Demographic and clinical characteristics of study population presented (1 star)<br>b) No description (No star)	a) Record linkage (1 star)<br>b) Self-report (No star)<br>c) No description (No star)	a) Assessment of ORP (1 star)<br>b) Assessment of SDF (1 star)<br>c) No description (No star)	a) Number of samples analyzed equal to the number of subjects in the group (1 star)<br>b) More than or equal to 80% of the sample analyzed or description for the exclusion provided (1 star)<br>c) Less than 80% of the sample analyzed and no description for the exclusion (No star)<br>d) No description (No star)	a) Fertile men/ donor group (1 star)<br>b) Infertile men (1 star)<br>c) No description (No star)<br>d) Others (No star)	
**Arafa, 2019**	1	1	1	1	1	2	1	1	9
**Arafa, 2020**	1	1	1	1	1	2	1	1	9

**Garcia-Segura, 2020**	1	1	0	0	1	2	0	1	6
**Gill, 2021**	1	1	1	1	1	2	0	1	8
**Homa, 2019**	1	1	1	0	1	2	0	1	7
**Majzoub, 2018**	1	1	1	1	1	2	1	2	10
**Tanaka, 2020**	1	1	0	1	1	2	1	1	8

Good quality: 2/4 stars for selection, 1/2 stars for comparability, 5/6 stars for outcome

Fair quality: 1/4 stars for selection, 1/2 stars for comparability, 4/6 stars for outcome

Poor quality: 0/4 stars for selection, 0/2 stars for comparability, 3/6 stars for outcome
